# The bromodomain inhibitor JQ1 up-regulates the long non-coding RNA *MALAT1* in cultured human hepatic carcinoma cells

**DOI:** 10.1038/s41598-022-11868-4

**Published:** 2022-05-11

**Authors:** Hae In Choi, Ga Yeong An, Eunyoung Yoo, Mina Baek, Bert Binas, Jin Choul Chai, Young Seek Lee, Kyoung Hwa Jung, Young Gyu Chai

**Affiliations:** 1 Department of Bionanotechnology, Hanyang University, Seoul, 04673 Republic of Korea; 2 Department of Molecular and Life Science, Hanyang University, Ansan, Gyeonggi-do 15588 Republic of Korea; 3 Institute of Natural Science and Technology, Hanyang University, Ansan, 15588 Republic of Korea; 4 College of Veterinary Medicine, Seoul National University, Seoul, 08826 Republic of Korea; 5 Convergence Technology Campus of Korea Polytechnic II, Incheon, 21417 Republic of Korea

**Keywords:** Cancer, Molecular biology

## Abstract

The epigenetic reader, bromodomain-containing 4 (BRD4), is overexpressed in hepatocellular carcinoma (HCC), and BRD4 inhibition is considered as a new therapeutic approach. The BRD inhibitor JQ1 is known to inhibit the enrichment of BRD4 at enhancer sites. Gene network analyses have implicated long non-coding RNAs (lncRNAs) in the effects of JQ1, but the precise molecular events remain unexplored. Here, we report that in HepG2 cells, JQ1 significantly reduced various proliferation-related lncRNAs, but up-regulated the known liver tumor marker, *MALAT1*. Using ChIP-sequencing data, ChIP-qPCR, luciferase reporter assays, and chromatin conformation capture (3C), we characterized the *MALAT1* gene locus. We found that JQ1 elicited a rearrangement of its chromatin looping conformation, which involved the putative enhancers E1, E2, E3, the gene body, and the promoter. We further found that the forkhead box protein A2 (FOXA2) binds to E2 and the promoter; suppression of FOXA2 expression resulted in *MALAT1* up-regulation and increased cell proliferation. These results suggest that the inhibition of *MALAT1* may improve the effect of BET inhibitors as an anti-cancer therapy and that FOXA2 would be a suitable target for that approach.

## Introduction

Hepatocellular carcinoma (HCC) is the most common type of primary liver cancer. HCC is prevalent cancer globally and a leading cause of cancer-related death^1,2^. Significant epigenetic alteration exists in HCC^3^. Therefore, epigenetic transcriptional regulators may be considered as potential therapeutic targets for anti-cancer treatment^4^. The epigenetic reader, BRD4, a member of the bromodomain and extraterminal (BET) proteins (BRD2, BRD3, BRD4, and BRDt) family, recognizes acetylated lysine residues of H3 tails with two tandem bromodomains (BD1 and BD2). Accumulation of BRD4 in hyper-acetylated chromatin regions, promoters, and enhancers facilitates their interaction and activates transcription^5^. In HCC, BRD4 is overexpressed and promotes gene expression related to cell migration, invasion, and apoptosis^6,7^. For example, BRD4 is closely associated with the overexpression of the key oncogene *MYC*; thus, inhibition of BRD4 is considered as a therapeutic strategy^8–10^. JQ1, a pan-bromodomain inhibitor with a high affinity to BRD4, enables the study of the antitumor effect of BRD4 inhibition^11,12^. Previous studies showed that JQ1 inhibits cancer cell proliferation and promotes apoptosis in various cancer cells by inhibiting BRD4 binding to super-enhancers of target genes^13^. Several studies were performed on transcriptome analysis to identify mechanisms and potential targets of BET inhibitors in the treatment of cancer^13^. More generally, the inhibition of BET proteins has been highlighted as a new therapeutic strategy for cancer, neurological, and inflammatory disease^14,15^

lncRNAs play diverse roles in regulating gene transcription, translation, post-transcriptional, and epigenetic modification^16^. Notably, lncRNAs play a role in tumor suppression (e.g., *GAS5, LINC-PINT, MEG3*) and tumorigenesis (e.g., *HOTAIR, RCAR4, MALAT1*). The abnormal expression of lncRNAs affects the malignity, growth, proliferation, and migration of cancer cells^17^. Thus, a role for lncRNAs in cancer has been established. However, the underlying mechanisms are poorly understood. Most reports are limited to genetic changes, mainly related to *MYC*^18,19^, while epigenetic mechanisms have received comparatively less attention. Here, we explored the mechanism of tumor-related lncRNA expression by inhibiting the BET protein, BRD4, in HepG2 cells, an established model for HCC.

## Results

### JQ1 treatment leads to the upregulation of *MALAT1*

**Figure 1:**
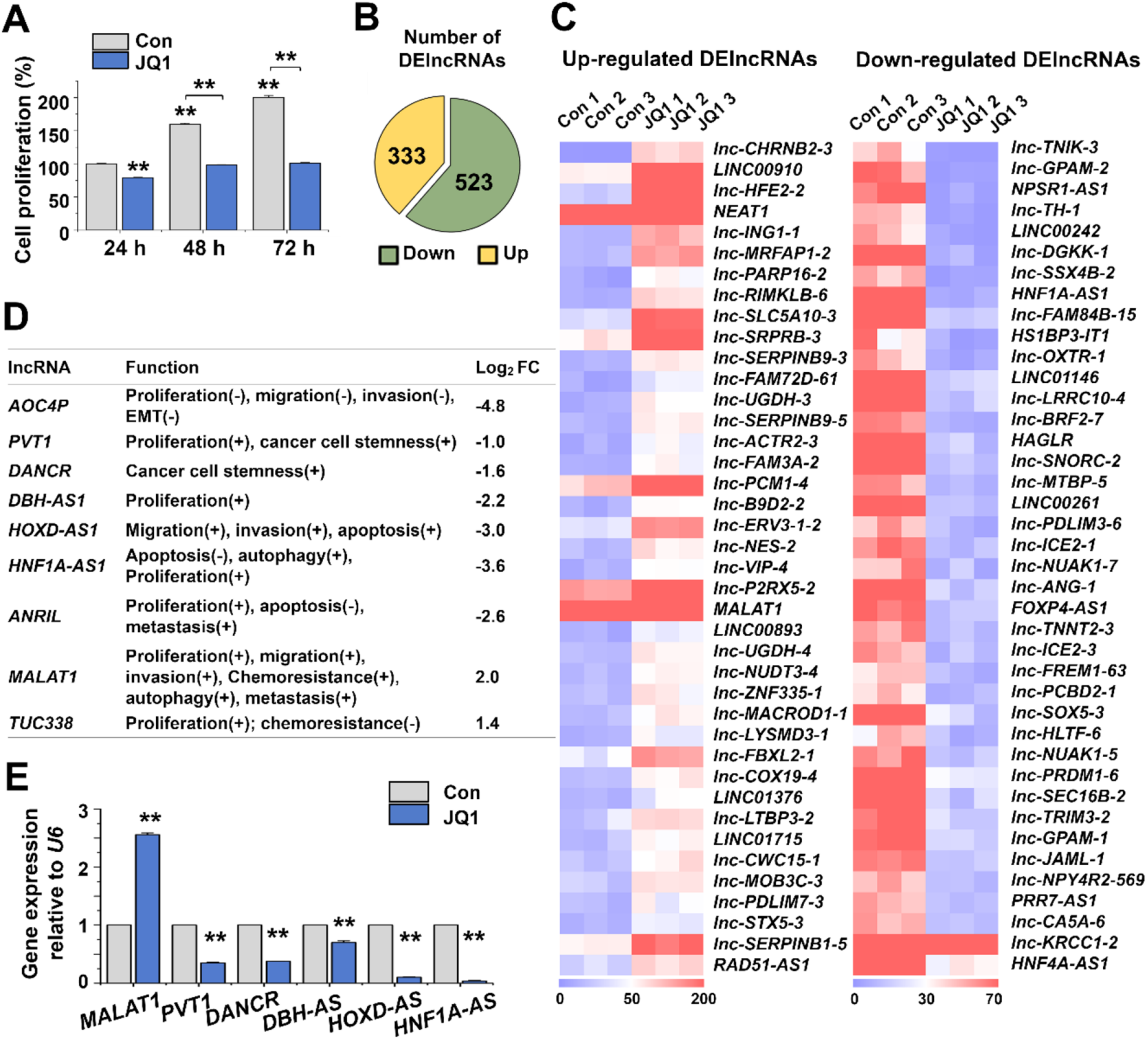
Differential lncRNA expression in JQ1-treated HepG2 cells. (**A**) HepG2 cells were treated with JQ1 (5 µM) or vehicle (DMSO) for the indicated durations, and cell proliferation was determined using a WST-1 assay. The data represent three biological indenpendent experiments. **p < 0.01. (**B**) Pie chart displaying the number of up-regulated (yellow) and down-regulated (green) lncRNAs. (**C**) Heat map representing the top 40 up- and down-regulated DElncRNAs. (**D**) Log_2_ fold changes of 9 selected lncRNAs affected by JQ1 and previously known to be over-activated in HCC. (**E**) qRT-PCR analysis of 6 selected DElncRNAs levels. The data represent three independent experiments. The values are mean ± SD of triplicate wells. **p < 0.01.

To study the role of BRD4 in the HepG2 cells, we treated them with JQ1. This led to a significantly reduced proliferation within 24 h, and the effect increased further until at least 72 h (Fig. 1A; Supplementary Fig. S1A). We used the 24 h-time point for RNA-seq analysis. Of a total of 856 differentially expressed lncRNAs (DElncRNAs), 333 were up-regulated and 523 down-regulated by JQ1 (Fig. 1B). Heatmaps of the top 40 up- and down-regulated DElncRNAs are shown in Fig. 1C (numerical values are listed in Supplementary Table S1).

At least some of the downregulated lncRNAs (Fig. 1D, E) were previously found to be highly expressed in liver cancer and to promote proliferation and metastasis (*AOC4P*, *PVT1, DANCR, DBH-AS1, HOXD-AS1, HNF1A-AS1, ANRIL*)^20–24^. These lncRNAs probably are also important for HepG2 cells: When we randomly subjected one of them (*DANCR*) to RNA interference (Supplementary Fig. S2A), this resulted in a markedly decreased number and proportion of EdU-positive HepG2 cells (Supplementary Fig. S2B,C), in line with the known oncogenic role of *DANCR*.

**Figure 2:**
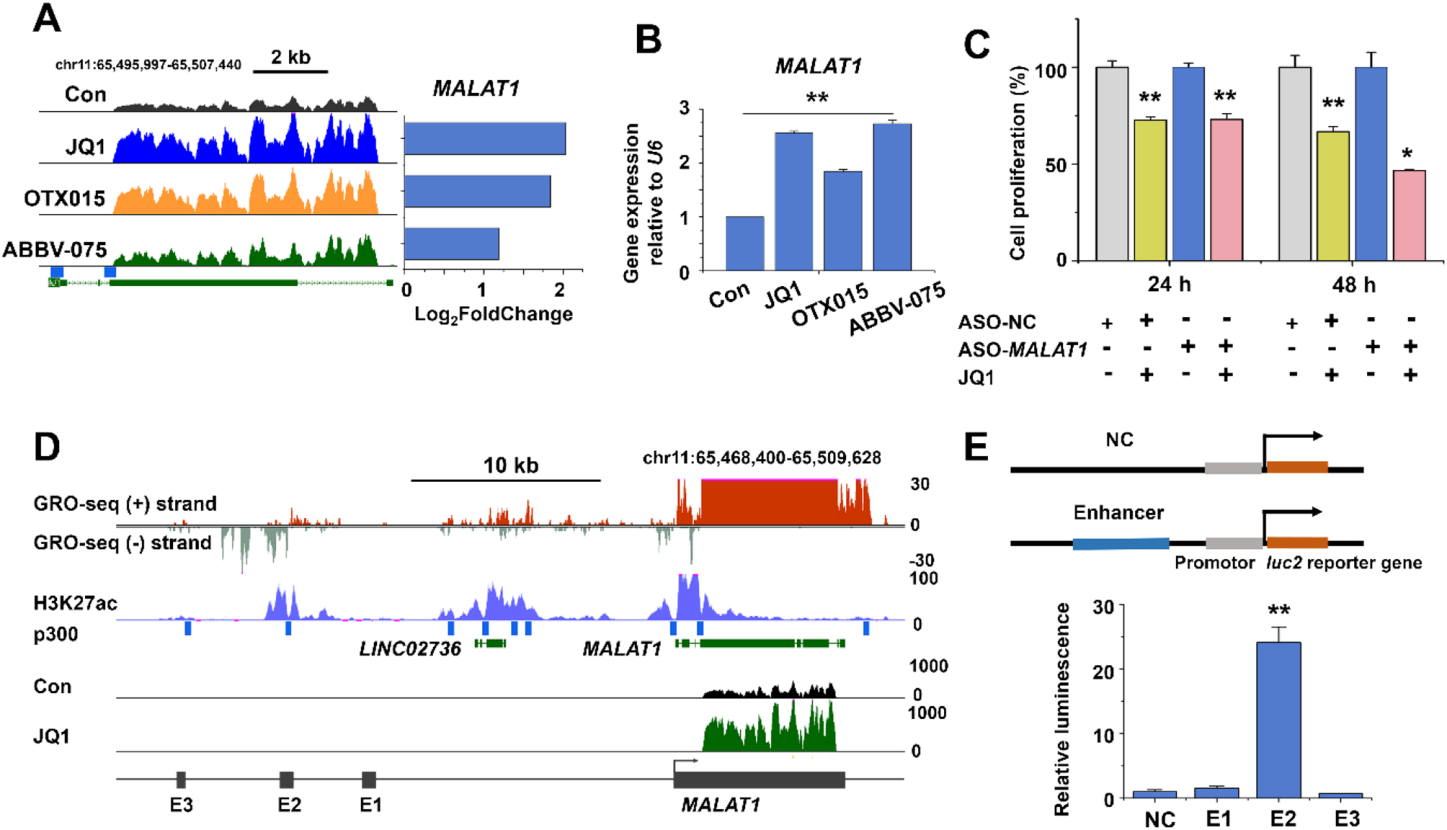
*MALAT1* expression and putative *MALAT1* enhancers in JQ1-treated HepG2 cells. (**A**) RNA-seq read densities (left) and corresponding log_2_ fold changes (right) of the *MALAT1* gene transcripts in BET inhibitor-treated vs. control HepG2 cells. (**B**) qRT-PCR analysis of *MALAT1* levels. The data represent three independent experiments. The values are mean ± SD of triplicate wells. **p < 0.01. (**C**) Cell proliferation was determined using WST-1 assay in *MALAT1* ASO- and/or JQ1-treated HepG2 cells. The data represent three biologically independent experiments. *p < 0.05 and **p < 0.01. (**D**) USCS genome browser view of the GRO-seq peaks, H3K27ac enrichment, p300 binding sites, and BRD4 binding sites along the *MALAT1* locus (chr11: 65,468,400–65,509,628). The potential *MALAT1* enhancer regions E1, E2, and E3 upstream of the *MALAT1* gene are denoted. *MALAT1* expression from RNA-seq read densities is represented with black (untreated) and green (JQ1-treated) peaks. (**E**) Verification of putative *MALAT1* enhancers by luciferase reporter gene assays. The data represent three independent experiments. **p < 0.01.

In contrast, we could not make an obvious physiological link for an up-regulated lncRNA (Fig. 1D, E; *MALAT1* and *TUC338*). Interestingly, one of them was *MALAT1* (Fig. 2A, B), which appeared paradoxical because *MALAT1* is known to be highly expressed in liver cancer^25^, in line with our own bioinformatics analysis using The Atlas of non-coding RNA in Cancer (TANRIC; https://ibl.mdanderson.org/tanric/design/basic/main.html)^26^ (Supplementary Fig. S3). However, the stimulation of *MALAT1* expression was observed not only with JQ1 but also with other BET inhibitors (OTX015 and ABBV-075) (Fig. 2A, B, obtained by RNA-seq and qRT-PCR, respectively). Furthermore, an antisense oligonucleotide (ASO) directed against *MALAT1* (Supplementary Fig. S4) increased the anti-proliferative effect of JQ1, although the oligo alone did not affect cell proliferation (Fig. 2C). This result indicated that the up-regulation of *MALAT1* dampened the anti-proliferative effect of JQ1 (Fig. 1A). We, therefore, decided to take a closer look at the *MALAT1* gene regulation in JQ1-treated HepG2 cells.

### Identification of putative *MALAT1* enhancers

We examined ENCODE ChIP-seq and global run-on sequencing (GRO-seq) data to localize the potential *MALAT1* enhancers (Fig. 2D). Using the GRO-seq peaks (GSE92375), H3K27ac ChIP-seq peaks (GSE29611), and p300 ChIP-seq peaks (GSE32465) at the UCSC Genome browser, we analyzed the upstream regions of *MALAT1*. In region (chr11: 65,487,241–65,488,714), we found enrichment for H3K27ac that co-localized with the lncRNA gene *LINC02736*, whose expression was decreased by JQ1 (Supplementary Fig. S5). In addition, we identified three putative enhancer loci (E1, E2, and E3) further upstream (Fig. 2D; Table 1). We observed an approximately 20-fold increased luciferase reporter gene expression by the E2 region but not the E1 or E3 regions (Fig. 2E). From these results, we hypothesized that the increased *MALAT1* expression in JQ1-treated HepG2 cells might be regulated by enhancer E2.

**Table 1:** *MALAT1* putative enhancer regions.

Putative enhancer	h38_DNA range
E1	chr11:65,481,488–65,482,092
E2	chr11:65,477,162–65,477,840
E3	chr11:65,471,480–65,471,854

### FOXA2, but not FOS, is involved in *MALAT1* expression and HepG2 cell proliferation

**Figure 3:**
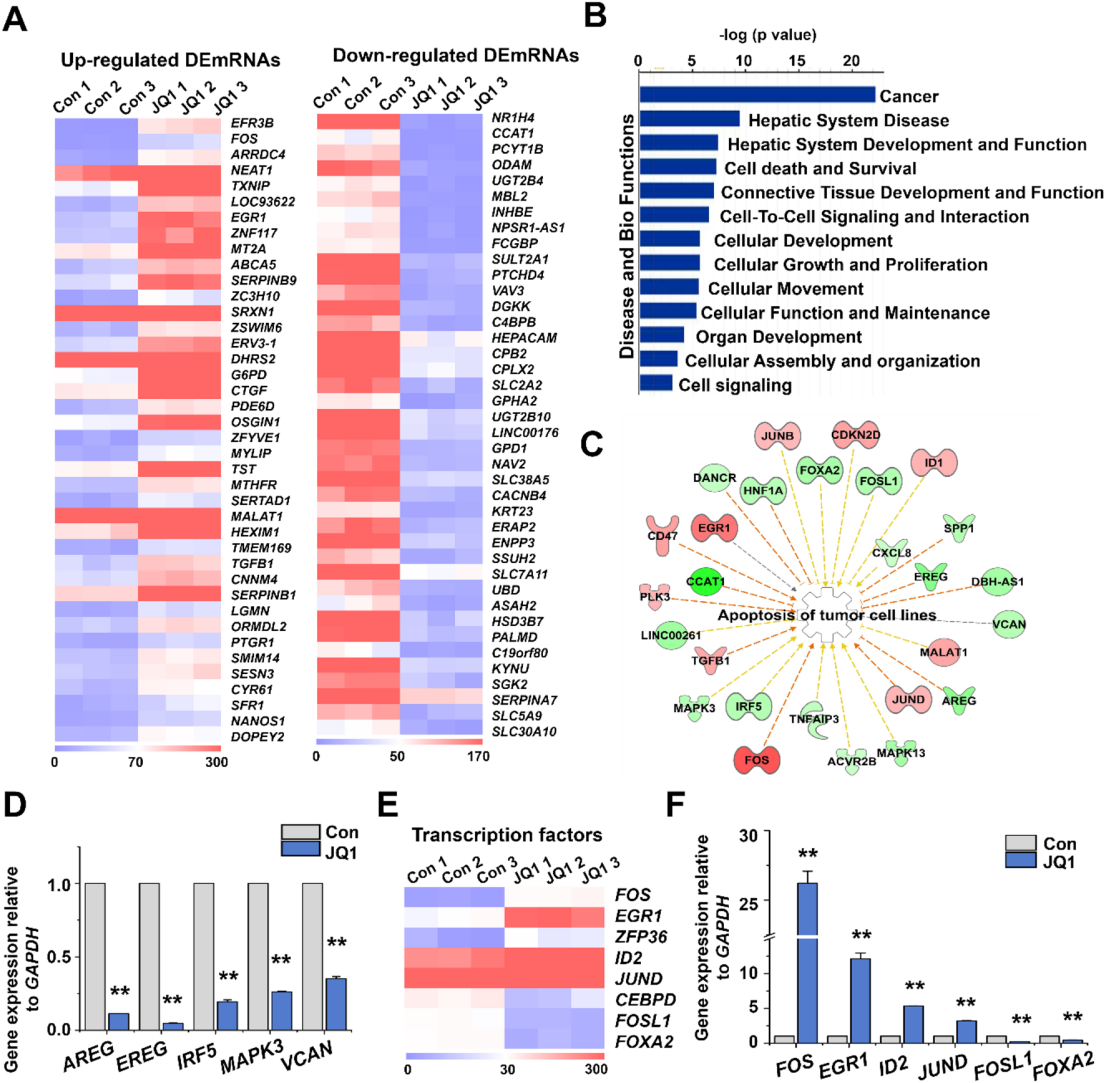
Expression of DEmRNAs and selected TFs in JQ1-treated vs. control HepG2 cells. (**A**) Heat map representing the top 40 up- and down-regulated DEmRNAs (*p-*value < 0.05, log_2_-fold change ≥ 1.5, log_2_-fold change ≤  − 1.5). (**B**) Disease and biofunction analysis of differentially expressed genes using IPA. (**C**) IPA network analysis of tumor cell apoptosis-related genes in JQ1-treated cells. The DEmRNAs are colored according to their predicted activation state following JQ1 treatment, activated (red) or suppressed (green). The arrows indicate predicted relationships: Red leads to activation; yellow, findings inconsistent with the state of downstream molecule. (**D**) qRT-PCR analysis of selected DEmRNAs. The data represent three independent experiments. The values are the mean ± SD of triplicate wells. **p < 0.01. (**E**) Heat map showing expression of eight selected TF mRNAs in JQ1-treated vs. control cells, each in triplicate. (**F**) Effect of JQ1 on the mRNA levels of FOXA2 and other TFs (qRT-PCR). The values are the mean ± SD of triplicate wells. **p < 0.01.

Next, we searched for potential regulators, especially transcription factors (TFs), that might be involved in the JQ1-caused *MALAT1* gene upregulation. Using RNA-seq, we found that 274 mRNAs were up-regulated and 737 down-regulated by JQ1 (Supplementary Fig. S6A). The heatmaps of the top 40 up- and down-regulated differentially expressed mRNAs (DEmRNAs) are shown in Fig. 3A (numerical values are listed in Supplementary Table S2). The DEmRNAs were associated with cancer, hepatic system disease, cell death and survival, and cellular growth and proliferation (Fig. 3B). Many down-regulated genes were related to angiogenesis and negative regulation of apoptosis (Supplementary Fig. S6B). IPA network analysis highlighted known tumor cell apoptosis-related genes (Fig. 3C), some of which we validated by qRT-PCR (Fig. 3D). More to the point, we found that several TFs were also altered, including the apoptosis-associated genes of Fig. 3C (Fig. 3E). Of these, we validated four up-regulated (*FOS, EGR1, ZFP36, ID2, JUND*) and two down-regulated (*FOSL1* and *FOXA2*) TFs by qRT-PCR (Fig. 3F).

**Figure 4:**
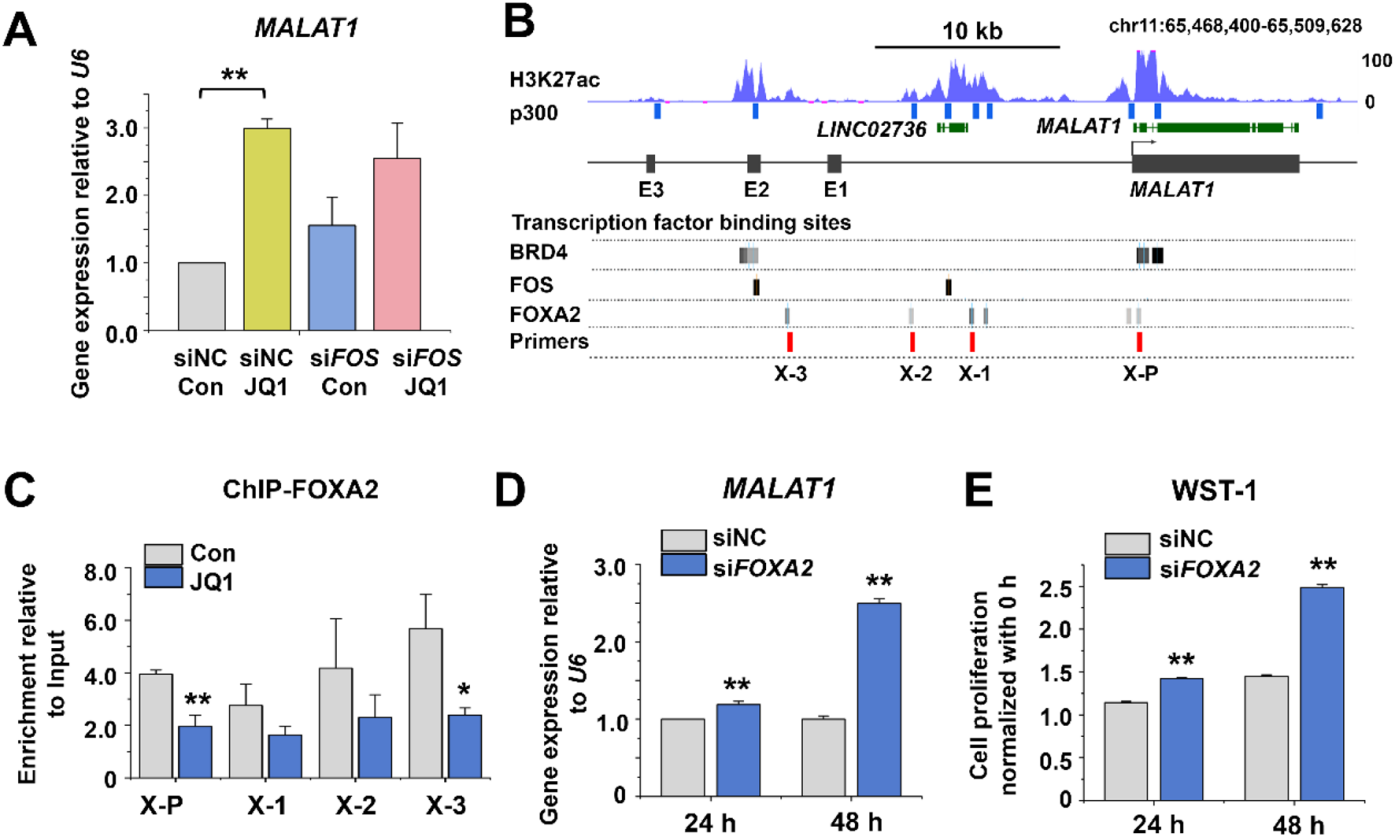
Effects of FOXA2 on *MALAT1* transcription and proliferation. (**A**) qRT-PCR analysis of *MALAT1* levels in JQ1- and *FOS* siRNA-treated HepG2 cells. The values are the mean ± SD of triplicate wells. **p < 0.01. (**B**) Analysis of FOS (GSM2797520) and FOXA2 (ENCSR066EBK) binding to the *MALAT1* locus. H3K27ac enrichment and TFs binding (gray lines) from published ChIP-seq data in HepG2 cells. (**C**) ChIP-qPCR analysis of FOXA2 binding in JQ1-treated HepG2 cells. X-P, X-1, X-2, X-3, amplicons (**B** red lines). Enrichment was calculated relative to input DNA from three independent experiments. The values are the mean ± SD of triplicate experiments. *p < 0.05 and **p < 0.01. (**D**) qRT-PCR analysis of *MALAT1* levels in *FOXA2* siRNA-treated cells. The values are the mean ± SD of triplicate wells. **p < 0.01. (**E**) Cell proliferation assay of *FOXA2* siRNA-treated cells. The data represent three biologically independent experiments. **p < 0.01.

Bioinformatics analysis (by IPA) suggested that one of the upregulated TFs, FOS, regulates *MALAT1* (Supplementary Fig. S7A), in line with DNA sequence analysis that revealed the co-localization of FOS binding sites and putative *MALAT1* enhancers (Supplementary Fig. S7B). However, both in the absence and presence of JQ1, the levels of *MALAT1* were not significantly changed by a *FOS* siRNA, neither was the JQ1-caused increment of *MALAT1* expression (Fig. 4A; Supplementary Fig. S7C). Furthermore, JQ1 did not increase the binding of FOS to the promoter and putative enhancer regions of *MALAT1* (Supplementary Fig. S7D). These results indicate that contrary to expectation, FOS is not involved in the regulation of *MALAT1* in the HepG2 cells.

Next, we focused on the down-regulated TF, FOXA2 (Fig. 3E, F). Bioinformatics analysis of published HepG2 ChIP-seq data indicates that FOXA2 binds to the putative enhancer E2 (X-3) and the promoter (X-P) regions of the *MALAT1* gene (Fig. 4B), as validated by our ChIP-qPCR analysis. These bindings were significantly reduced by JQ1 (Fig. 4C). In contrast, the binding of FOXA2 to X-1 and X-2 did not co-localize with E1 or E3 (Fig. 4B), and the JQ1 treatment did not elicit a statistically significant change of FOXA2 binding to X-1 and X-2 (Fig. 4C). These results suggest that the direct binding of FOXA2 to the *MALAT1* promoter and enhancer E2, but not E1 or E3, interferes with the transcription of *MALAT1*, thus mirroring the effect of E2, but not E1 or E3, on luciferase reporter gene expression (compare with Fig. 2E).

The reduction of FOXA2 mRNA (Supplementary Fig. S7E) and protein (Supplementary Fig. S7F) by RNA interference led to a significant increase of *MALAT1* expression (Fig. 4D) and an increase in the proliferation of the HepG2 cells (Fig. 4E). Of note, we observed the same reciprocal relationship between FOXA2 and *MALAT1* in Huh7 cells, another human HCC line (Supplementary Fig. S8). This result suggests that *MALAT1* expression stimulates cell proliferation under negative control by FOXA2.

### JQ1 treatment reconfigures the *MALAT1* locus

**Figure 5:**
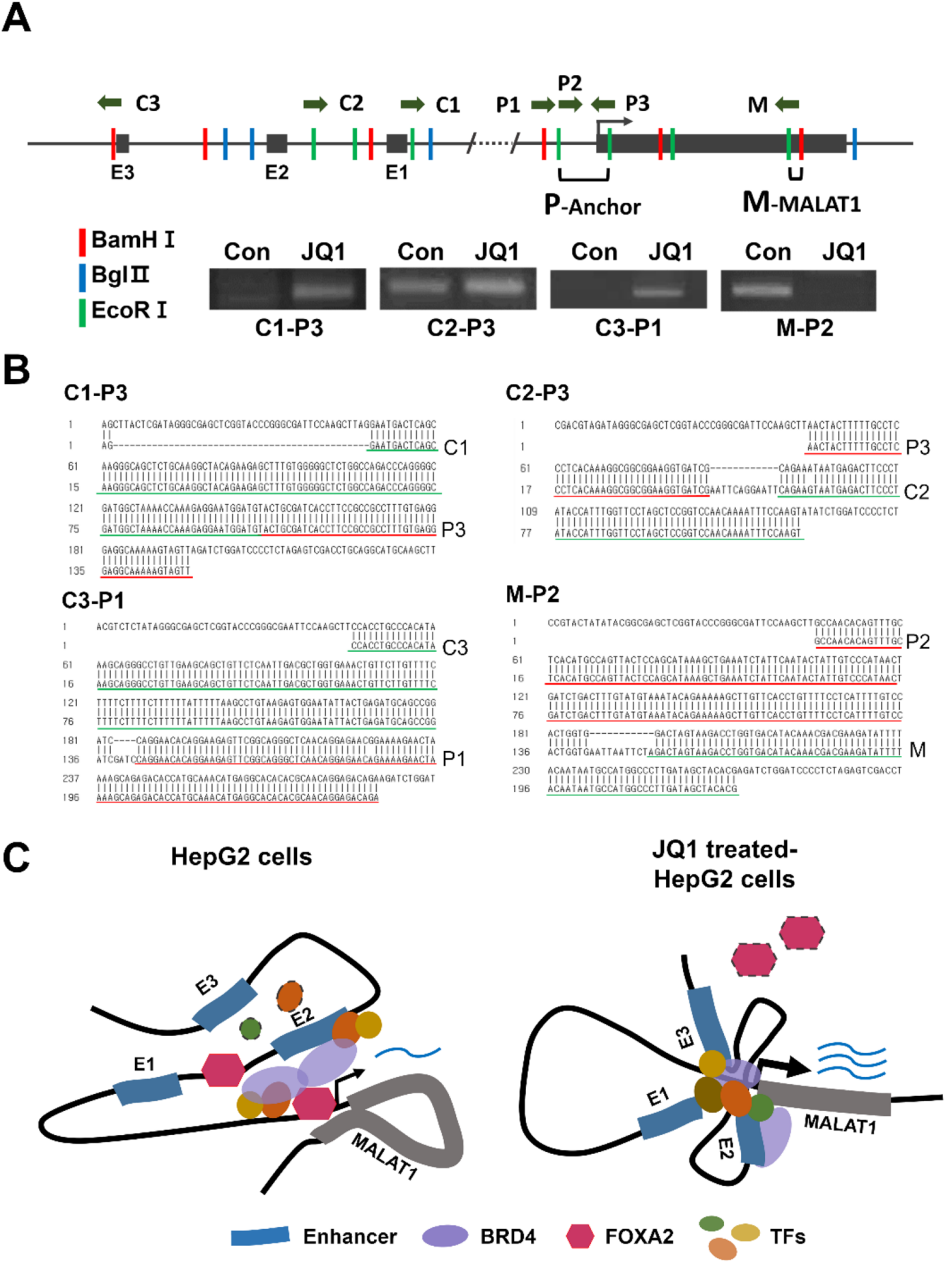
JQ1 reconfigures the *MALAT1* gene locus. (**A**) 3C analysis of *MALAT1* gene locus in JQ1-treated and control HepG2 cells. Top, diagram of the *MALAT1* gene locus showing the restriction enzyme sites used for 3C. Bottom, PCR results of 3C experiment. E1, E2, E3, putative enhancers; M, P1, P2, P3, C1, C2, C3, primers. (**B**) Sequencing of the *MALAT1* enhancer and promoter intrachromosomal loop products. Green and red lines indicate the sequences of each fragment marked on the right. (**C**) Schematic interpreting the 3C analysis. In untreated HepG2 cells (left), the E2 enhancer and FOXA2 and BRD4 are key components of the promoter-associated chromatin complex, in contrast with E1 and E3. In JQ1-treated HepG2 cells (right), the reconfigured chromatin complex also involves E1 and E3 but loses FOXA2 and a significant portion of the BRD4. These changes result in an increased expression of *MALAT1*.

To better understand the mechanism of how JQ1 affects *MALAT1* expression, we performed a 3C assay. Using the promoter region as the anchor (P), we assessed the relative positions of E1, E2, and E3 in the absence and presence of JQ1. Figure 5A shows that in the absence of JQ1, E2 (amplicon C2-P3) and the gene body M (amplicon M-P2), but neither E1 (amplicon C1-P3) nor E3 (amplicon C3-P1), associated with the promoter. Upon the addition of JQ1, all three putative enhancers became associated with the promoter, while the gene body was no longer associated (Fig. 5A). These interactions were confirmed by sequencing the agarose gel bands (Fig. 5B).

## Discussion

This study found that when HepG2 cells were treated with JQ1, the long non-coding RNA *MALAT1*, which has been positively correlated with malignancy, was up-regulated. Our data suggest the down-regulation of the transcription factor FOXA2 and a reconfiguration of the associated chromatin complex as an underlying mechanism.

The JQ1-caused up-regulation of *MALAT1* appears paradoxical because BET inhibitors are being considered as anti-cancer agents. However, *MALAT1* is highly expressed in various cancers, including liver, lung, and breast cancer, and plays a role in cancer progression^27^. In addition, the *MALAT1* expression level is negatively correlated with the survival rate in cancer patients^28^. *MALAT1* induces cell proliferation and metastasis via the MAPK/ERK and PI3K/AKT signaling pathways in retinoblastoma and ovarian cancer, respectively^29,30^, and it is known to enable the high expression of the key oncogene *MYC* in thymic epithelial tumors^31^. Interestingly, in HepG2 cells, *MALAT1* was also found in mitochondria, and its knockdown limited ATP synthesis and tumor cell invasion^32^. In addition, *MALAT1* causes chemotherapy resistance by regulating miR-216b in HCC^23^. Taken together, literature strongly suggests that *MALAT1* expression should be considered as an undesired feature of HCC and other tumors.

We have recently shown that JQ1 down-regulates *MYC* in HCC cells^33^, which is in line with the anti-cancer effects of JQ1 in other tumors. Similarly, JQ1 reduced the expression of pro-apoptotic *BCL2L11* in HCC^9^. However, in prostate cancer, JQ1 inhibited the transcriptional repressor FOXA1, thereby increasing the expression of invasion genes^34^ or even activating the DNA damage response^35^. The increased expression of *MALAT1* after the JQ1 treatment that we described here may also contribute to the unwanted effects of JQ1. These findings collectively emphasize the need to learn more about the mechanisms of BET inhibitors as potential anti-cancer agents. Hence, investigating the mechanisms regulating the overexpression of *MALAT1* by JQ1 treatment may contribute to understanding the unwanted side effects of the BET inhibitors.

In our study, contrary to expectations^36^, the general TF FOS did not regulate *MALAT1*. Instead, we identified the lineage-specific TF FOXA2 as a candidate for the modulation of *MALAT1* expression in HepG2 cells. The forkhead box (FOX) proteins are transcription factors related to cancer development and progression. FOXA1 is a well-studied regulator of estrogen receptor (ER) and androgen receptor (AR) activity in breast and prostate cancer^37^. In this context, FOX proteins play a crucial role in the rearrangement and reprogramming of super-enhancers^38,39^. FOXA1 and FOXA2 regulate the transcription of liver-specific genes and are known to complement each other^40^. In addition, the importance of FOXA2, particularly concerning liver disease, has been demonstrated^41^. Interestingly, FOXA1 and FOXA2 play dual roles as tumor suppressors and oncogenes^42^. FOXA1 is a transcriptional repressor and reduces the viability and motility in liver cancer cells^43^, while FOXA2 inhibits EMT in HCC, breast cancer, and lung cancer^37,44,45^. Hence, our data suggest that a focus on FOXA2 in HCC may help address the problem of JQ1’s and potentially other BET inhibitors’ detrimental effects in anti-cancer therapy.

In the present study, we associated the JQ1-promoted *MALAT1* expression with decreased binding of FOXA2 to the promoter and E2 along with the formation of an (E1, E2, E3)-promoter complex, where E1, E2, and E3 are putative enhancers that we identified. JQ1 has been shown to directly bind to FOXA1, which neutralizes the repressor function of that TF^34^. Our finding that JQ1 reduced the binding of FOXA2 to the *MALAT1* gene locus, along with an increase of *MALAT1* expression, points to a similar mechanism.

Our data show that JQ1 affects *MALAT1* expression by two mechanisms. The first mechanism is indirect and is mediated by the reduced expression of *FOXA2*, probably caused by the interference of JQ1 with the activity of BRD4 at the *FOXA2* locus. This mechanism would be similar to the typical effects of JQ1 on other genes. It leads to the increased expression of *MALAT1*, as supported by our findings that FOXA2 binds to E2 and that a knockdown of *FOXA2* increased the expression of *MALAT1*. These data reveal that FOXA2 is a repressor of the *MALAT1* gene in the HepG2 cells. The second mechanism directly affects *MALAT1* expression, as indicated by our finding (by ChIP-qPCR) of a reduced association of BRD4 with the *MALAT1* promoter region upon JQ1 treatment. However, the outcome (stimulation versus inhibition of *MALAT1* expression) is not yet certain. In general, one might expect that the reduced BRD4 availability reduces the expression of *MALAT1* just like it reduces the expression of *FOXA2* and other genes. Such a mechanism would counteract the indirect, FOXA2-mediated effect. However, our 3C analysis of the *MALAT1* promoter and upstream region points to the opposite possibility. We found that JQ1 treatment, which implies a reduced BRD4 level, led to a re-organization of the enhancer-containing chromatin loops associated with the *MALAT1* promoter. We note that even reduced levels of BRD4/mediators by BET inhibitors are sufficient to maintain enhancer-promoter interaction^46^. In addition to E2 (now free of its repressor), the putative enhancers E1 and E3 became directly associated with the promoter, suggesting the possibility of a stimulatory effect on *MALAT1* gene expression. Future experiments will need to determine the direct effect of JQ1 on *MALAT1* gene expression and the relative contributions of the indirect vs. direct mechanisms. It is worth mentioning that we observed the reciprocal relationship between the FOXA2 and *MALAT1* also in the independently derived Huh7 human HCC cell line (Supplementary Fig. S8), indicating that the mechanistic relationships that we studied in the HepG2 cells are not a cell line-specific artifact.

In conclusion, our study suggests a regulatory model for the up-regulation of the lncRNA *MALAT1* due to JQ1 treatment (Fig. 5C). The model predicts that manipulating *MALAT1* expression could improve the therapeutic effect of BET inhibitors in HCC. Firstly, JQ1 inhibits the binding of FOXA2, a repressor of *MALAT1* expression, to the *MALAT1* enhancer E2 and the promoter. Secondly, alteration of chromatin looping recruits the enhancers E1 and E3 to the promoter site. Thus, further analysis of the *MALAT1* promoter-associated chromatin looping is likely to suggest additional approaches to improve the BET-based therapy.

## Experimental procedures

### Cell culture and BET inhibitor treatment

The HCC cell line HepG2 was purchased from the Korean Cell Line Bank. HepG2 cells were cultured in Minimum Essential Medium supplemented with 10% fetal bovine serum (FBS) and penicillin (100 units/ml)/streptomycin (100 mg/ml) (Thermo Fisher Scientific, Waltham, MA, USA). The medium was replaced every 3–4 days. The cells were cultured in a humidified incubator at 37 °C with a 5% CO_2_ atmosphere. JQ1 was purchased from MedChemExpress (Monmouth Junction, NJ, USA). JQ1 was present at a concentration of 5 μM for 24 h.

### Total RNA sequencing

RNA sequencing (RNA-seq) was performed as previously described^47^. Total RNA was extracted from HCC cells using RNAiso Plus (Takara, Shiga, Japan) and a Qiagen RNeasy Mini kit (Qiagen, Hilden, Germany). RiboMinus Eukaryote kit (Invitrogen, Carlsbad, CA, USA) was used for Ribosomal RNA (rRNA) depletion. An RNA library was created by a NEBNext Ultra directional RNA library preparation kit from Illumina (New England BioLabs, Ipswich, MA, USA). RNA library sequencing was performed on the Illumina HiSeq2500 platform (Macrogen, Seoul, Korea). Transcriptome sequencing was performed on independent RNA samples from DMSO-treated (3 samples) and JQ1-treated (3 samples) HepG2 cells in biological triplicate.

### Differentially expressed genes analysis using RNA-seq data

For mRNA analysis, FASTQ files from RNA-seq were clipped and trimmed of adapters, and low-quality reads were removed using Trimmomatic^48^. These FASTQ files were aligned using STAR (version 2.7.8) aligner software with a UCSC hg38 reference^49^. Differentially expressed mRNAs (DEmRNAs) were analyzed using DESeq2 with the default parameters^50^. For lncRNA analysis, the raw data were trimmed with Trimmomatic (version 0.36)^48^ and processed using Bowtie2 (version 2.3.5)^51^ or STAR (version 2.7.8)^49^ aligner software with a GenCode GRCh38 reference (https://www.gencodegenes.org/human/) or an LNCipedia reference (https://lncipedia.org/; version 5.2)^52^. RNAs that exhibited an absolute log_2_-fold change larger than 1.5 or smaller than − 1.5 (log_2_-fold change ≥ 1.5 and log_2_-fold change ≤ −1.5, *p*-adjusted < 0.05) were designated as DEmRNAs or DElncRNAs. The dataset accession number GSE158552 was deposited in the Gene Expression Omnibus database^53^.

### Gene and lncRNA expression analysis using quantitative reverse transcription-PCR (qRT-PCR)

Total RNA was extracted from HepG2 cells using RNAiso Plus (Takara, Shiga, Japan) according to the manufacturer’s instructions. cDNA was synthesized by PrimeScript reverse transcriptase (Takara, Shiga, Japan) and amplified using gene-specific primers (Supplementary Table S4). The primers were designed by BLAST (https://blast.ncbi.nlm.nih.gov/Blast.cgi). qRT-PCR was performed with TBGreen *Premix Ex Taq* II (Takara, Shiga, Japan). Glyceraldehyde-3-phosphate dehydrogenase (*GAPDH*) or RNU6-1 (*U6*) were used as an internal control. After performing qRT-PCR, the results were analyzed using the critical threshold (△C_T_) and the comparative critical threshold (△△C_T_) methods in ABI 7500 (Applied Biosystems, Foster City, CA, USA) software with the NormFinder and geNorm PLUS algorithms. The data represent three independent experiments (n = 3).

### Cell proliferation assay

Cell proliferation was assessed using a premixed water-soluble tetrazolium salt (WST-1) cell viability test (Takara, Shiga, Japan) according to the manufacturer's instructions. The cells were seeded at a density of 5 × 10^3^ cells per well and treated with JQ1 for different durations (0 h, 24 h, 48 h, and 72 h). WST-1 was added to each well. After an additional 4 h incubation, absorbances were measured at 450 nm. The data represent three independent experiments (n = 3).

Ethynyldeoxyuridine (EdU) analysis was performed using an EdU Cell Proliferation Assay kit (Invitrogen, CA, USA), following the manufacturer’s instructions. After that, the cells were washed with phosphate-buffered saline, mounted with a 4’,6-diamidino-2-phenylindole (DAPI)-containing mounting solution (Vectashield, Vector Laboratories, Burlingame, CA, USA), and imaged by microscopy (Nikon Eclipse 80i, Tokyo, Japan). The percentage of EdU-positive cells was assessed using ImageJ (Bethesda, MD, USA) software. The data represent three independent experiments (n = 3).

### Knockdown of gene expression using siRNA treatment

Knockdown (KD) of gene expression was performed using small interfering RNA (siRNA). After seeding, the cells were transfected with siRNA constructs and scrambled siRNAs using the RNAiMax transfection agent (Thermo Fisher Scientific, Waltham, MA, USA) according to the manufacturer’s instructions. *FOS* siRNA (si*FOS*-1 ID: 115631 and si*FOS*-2 ID: VHS41046), *DANCR* siRNA (si*DANCR*-1 ID: n505292 and si*DANCR* ID: n272702), *FOXA2* siRNA (si*FOXA2*-1 ID: s6691 and si*FOXA2*-2 ID: s6692), and Silencer Negative Control siRNA (AM4611) were purchased from Thermo Fisher Scientific. The siRNAs were used at a concentration of 10 nM for 48 h in the growth medium.

### Knockdown of *MALAT1* expression using ASO treatment

Knockdown (KD) of *MALAT1* gene expression was performed using locked nucleic acid (LNA)-modified antisense oligonucleotides (ASOs). After seeding the cells, transfection was performed using RNAiMax transfection agent according to the manufacturer’s instructions with ASO constructs and scrambled ASOs. *MALAT1* antisense LNA GapmeR and LNA GapmeR Negative control B were purchased from Qiagen. *MALAT1* siRNA and scrambled siRNA were used at 10 nM or 50 nM for 24 h or 48 h in the growth medium.

### Chromatin immunoprecipitation quantitative PCR (ChIP-qPCR)

The chromatin immunoprecipitation (ChIP) assay was performed as previously described^54^. Briefly, the HepG2 cell chromatin was incubated with antibodies against BRD4 (Bethyl; A301-985A50), FOS (SCBT; sc-166940x), FOXA2 (Abcam; ab256493) and then precipitated with Dynabeads Protein A beads (Invitrogen, CA, USA); normal rabbit IgG (CST; 2729) and normal mouse IgG (Santa Cruz; sc-2025) were used as controls. The immunoprecipitated DNA was analyzed by qRT-PCR, and the expression levels were normalized to the amounts of input DNA. The data represent three independent experiments (n = 3). Primers used for ChIP-qPCR are listed in Supplementary Table S5.

### Genomic data analysis

We re-analyzed public H3K27ac ChIP-sequencing (seq) data sets in Gene Expression Omnibus (GEO) (GSE29611) as described previously^55^ and global run-on sequencing (GRO-seq) data sets in GEO (GSE92375). For the re-analysis, Trimmomatic (version 0.36)^48^ was used to trim the raw data and processed using Bowtie2 (version 2.3.5)^51^ or STAR (version 2.7.8)^49^ aligner software with a UCSC hg 38 reference. The ChIP-seq and GRO-seq peaks identified were analyzed with Homer (version 4.11)^56^ and visualized using UCSC Genome Browser (https://www.genome.ucsc.edu).

### Western blotting assay

Cells were lysed with RIPA buffer for protein extraction after treatment. Proteins were separated using sodium dodecyl sulfate (SDS) polyacrylamide gel electrophoresis (SDS-PAGE) and transferred to polyvinylidene difluoride membranes (Schleicher & Schuell Bioscience, Inc., Keene, NH, USA). The western blotting assay was performed using anti-β-actin (SCBT; sc-8432) and anti-FOXA2 (Abcam; ab256493) antibodies, both diluted at 1:1000.

### Luciferase reporter assay

Putative enhancer regions (E1, E2, and E3) were amplified with LongAmp *Taq* 2X Master Mix (New England Biolabs, Ipswich, MA, USA), using forward and reverse primers that generated NheI and XhoI sites, respectively. These amplicons were cloned into the pGL4.26 construct (Promega, Madison, WI, USA). The primers used for cloning are listed in Supplementary Table S3. The cells were seeded into 24-well plates and transfected with Lipofectamine 3000 (Thermo Fisher Scientific, Waltham, MA, USA). Luciferase activity was measured using the Dual-Glo Luciferase Assay kit (Promega, Madison, WI, USA). PRL-TK (*Renilla* luciferase expression construct; Promega) was used as an internal control. Luciferase activity was normalized to *Renilla* luciferase and the control (empty vector).

### Chromosome conformation capture assay

Chromosome conformation capture (3C) assay was performed as previously described, with minor modifications^57^. HepG2 cells were cross-linked with 1% formaldehyde, and nuclei were prepared from approximately 1–2 × 10^6^ cells. Five hundred units of BamHI, BglII, and EcoRI were used to digest the DNA overnight, followed by ligation and purification. The 3C products were quantified by Qubit assay kits (Thermo, Q32851) and amplified by PCR using TB Green *Premix Ex Taq* (Takara, BR420). The ligation of fragments was analyzed using agarose gel electrophoresis. Sequences of primers are presented in Supplementary Table S6.

### Statistical analysis

Data are presented as the mean ± standard deviation (SD) of the mean. All statistical analyses were performed using the IBM SPSS Statistics 26.0 program (IBM corp., Armonk, NY). We used a one-way analysis of variance followed by Tukey’s honestly significant difference post hoc test. *p-*values < 0.05 were considered significant.

## Supplementary Information

Supplementary Information.

## Data Availability

The raw data of RNA-sequencing were deposited in the Gene Expression Omnibus (GEO) database with accession number GSE158552^51^.

## Abbreviations

HCC: Hepatocellular carcinoma
DEmRNAs: Differentially expressed mRNAs
DElncRNAs: Differentially expressed lncRNAs
GRO-seq: Global run-on sequencing
H2K27ac: Acetylated H3 lysine 27
Chr: Chromosome
qRT-PCR: Quantitative reverse transcription-polymerase chain reaction
3C: Chromatin conformation capture
