# Supplementary material for: The bromodomain inhibitor JQ1 up-regulates the long non-coding RNA MALAT1 in cultured human hepatic carcinoma cells
Source: Sci Rep. 2022 May 11;12:7779. doi: 10.1038/s41598-022-11868-4 (PMC9095596; doi:10.1038/s41598-022-11868-4)
Supplement: Supplementary file 1 — Supplementary Information. [file 41598_2022_11868_MOESM1_ESM.docx]

**The Bromodomain Inhibitor JQ1 Up-regulates the Long Non-Coding RNA *MALAT1* in Cultured Human Hepatic Carcinoma Cells**

Hae In Choi^1^, Ga Yeong An^1^, Eunyoung Yoo^1^, Mina Baek^2, 3^, Bert Binas^2^, Jin Choul Chai^4^, Young Seek Lee^4^, Kyoung Hwa Jung^5*^, Young Gyu Chai^1,2*^

^1^ Department of Bionanotechnology, Hanyang University, Seoul, 04673, Republic of Korea.

^2^ Department of Molecular & Life Science, Hanyang University, Ansan, 15588, Republic of Korea

^3^ Institute of Natural Science and Technology, Hanyang University, Ansan, 15588, Republic of Korea.

^4^ College of Veterinary Medicine, Seoul National University, Seoul, 08826, Republic of Korea

^5^Convergence Technology Campus of Korea Polytechnic II, Incheon, 21417, Republic of Korea.

* Corresponding authors

**Supplementary Figures**


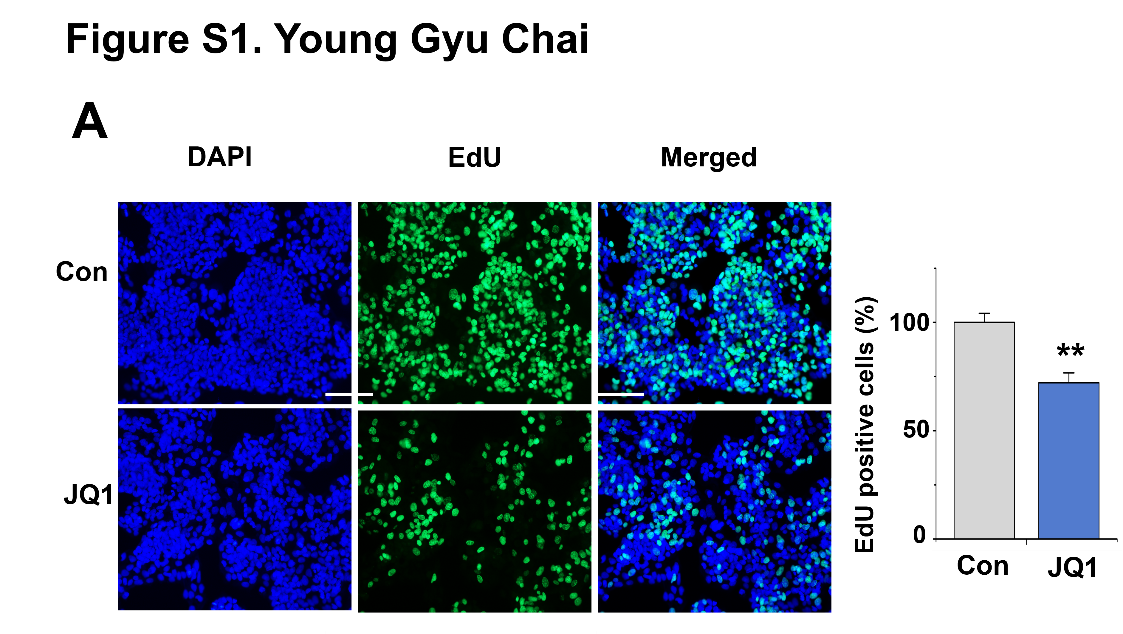


**Figure S1. The effects of JQ1 treatment on cell proliferation**

**A.** HepG2 cells treated with 5 μM of JQ1 for different durations (24 h, 48 h, and 72 h). Cell proliferation was determined using EdU assay. The data represent three biologically independent experiments. **, *p* < 0.01.


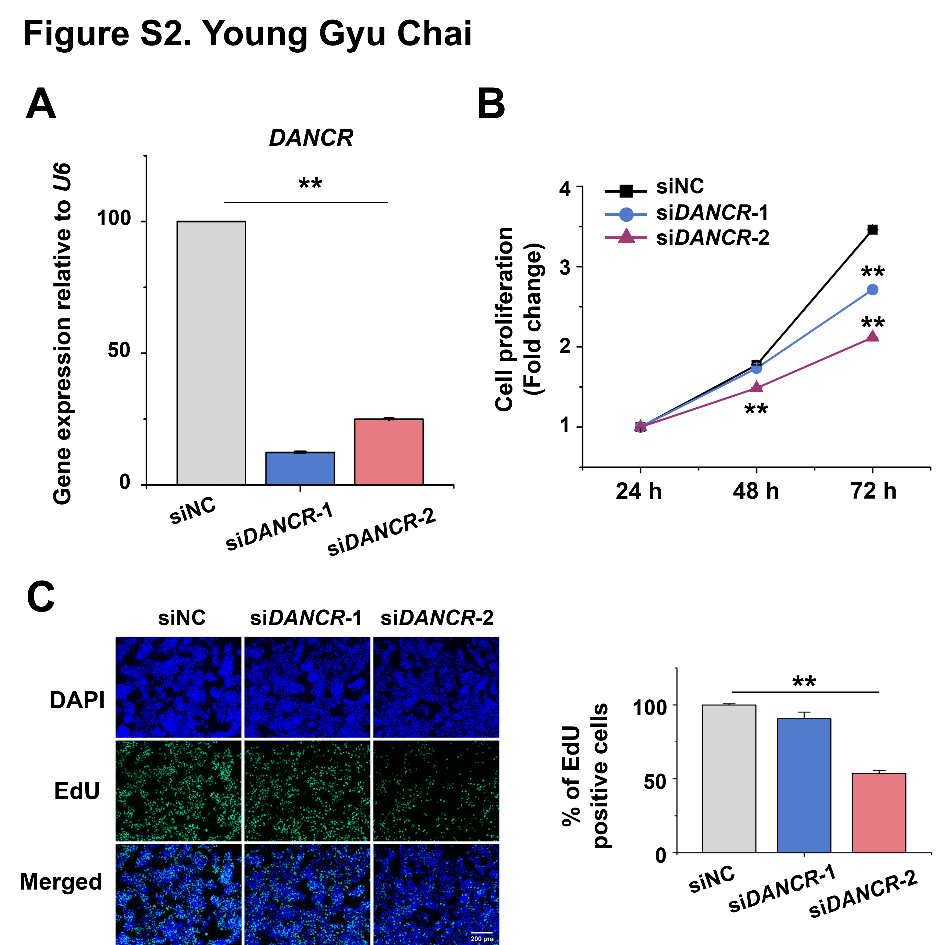


**Figure S2. The effects of lncRNA *DANCR* inhibition on cell proliferation**

**A.** Attenuation of lncRNA *DANCR* was performed using small interfering RNA (siRNA). The data represent three independent experiments. The values are the mean ± SD of triplicate wells. **, *p* < 0.01. **B and C.** Effect of siRNA treatment directed against *DANCR*. Cell proliferation was determined using a WST-1 assay (B) and EdU assay (C)**.** The data represent three biologically independent experiments. **, *p* < 0.01.

**
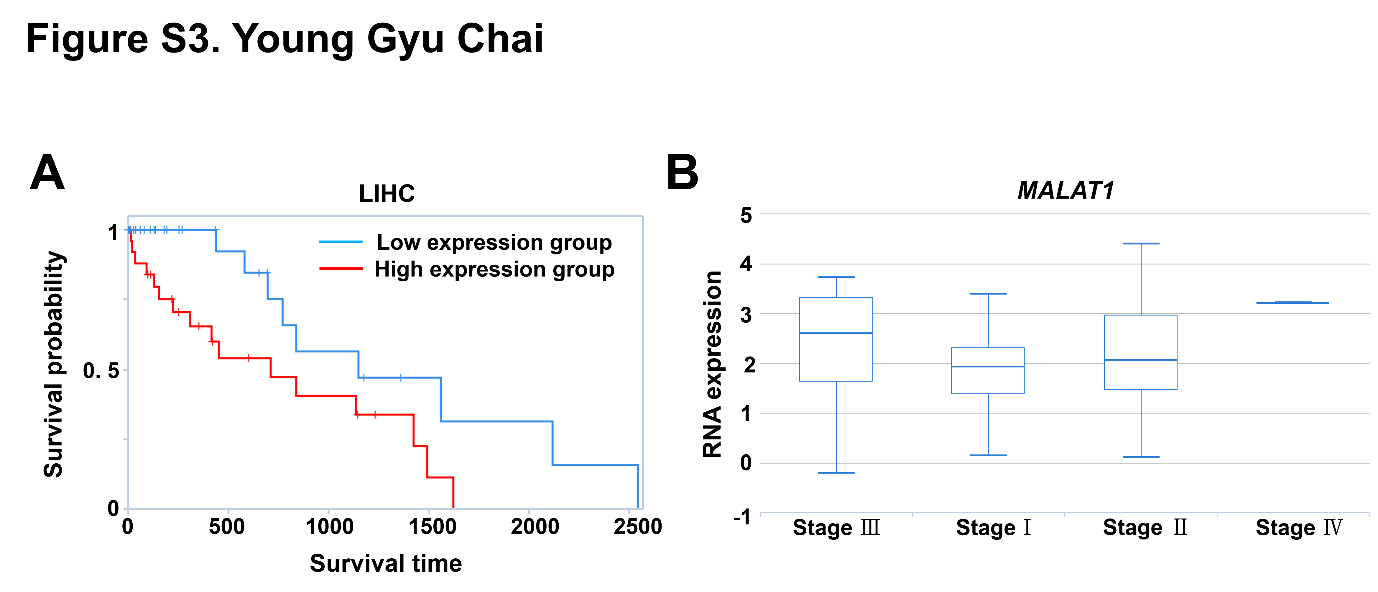
**

**Figure S3. *MALAT1* expression is correlated with a reduced survival probability of LIHC patients**

**A.** Kaplan-Meier plots derived from TANRIC depict significant overall poor survival of TCGA liver hepatocellular carcinoma (LIHC) patients associated with high expression levels of *MALAT1*. **B.** Data collected from TCGA shows the relative expression of *MALAT1* according to cancer stage.


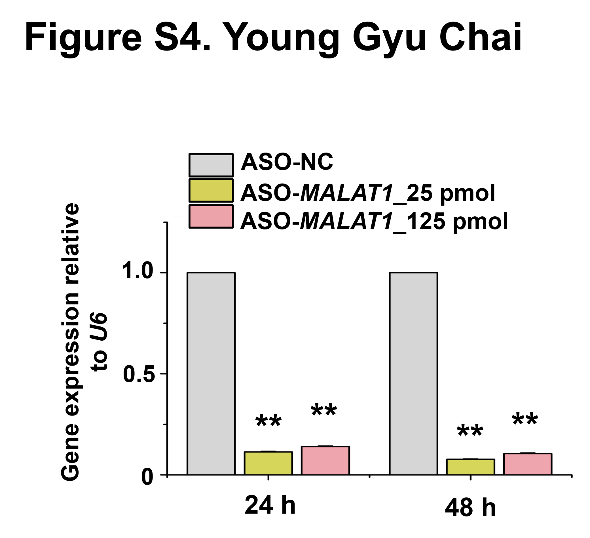


**Figure S4. Inhibition of *MALAT1* by antisense oligonucleotides**

qRT-PCR analysis verifying reduced *MALAT1* levels in HepG2 cells treated with locked nucleic acid (LNA)-modified antisense oligonucleotides. The data represent three independent experiments. The values are the mean ± SD of triplicate wells. **, *p* < 0.01.


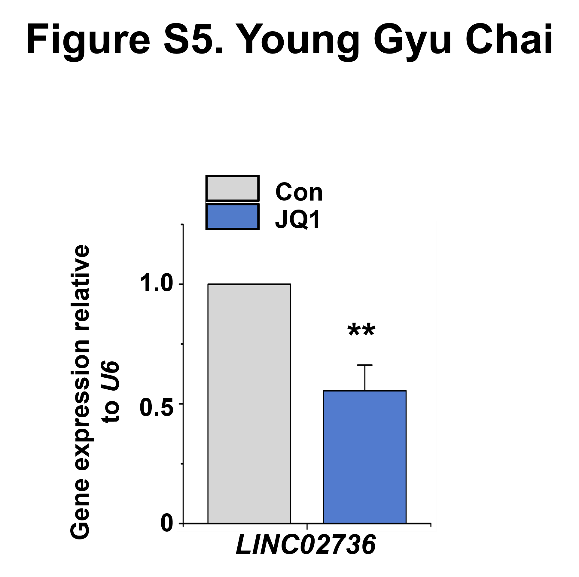


**Figure S5. *LINC02736* expression in JQ1-treated HepG2 cells.**

Effect of JQ1 on *LINC02736* expression (qRT-PCR). The data represent three independent experiments. The values are the mean ± SD of triplicate wells. **, *p* < 0.01.

**
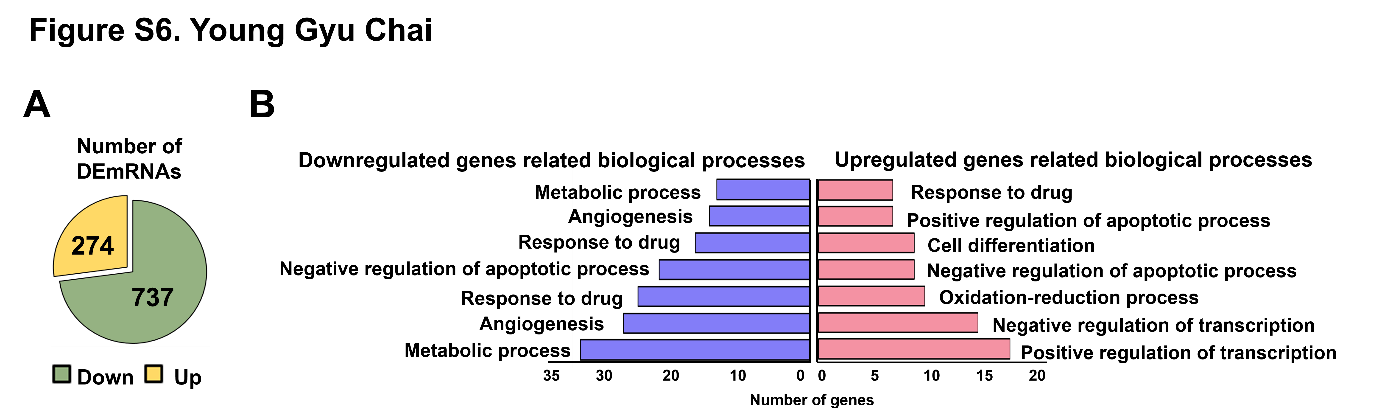
**

**Figure S6. Analysis of DEmRNAs in JQ1-treated HepG2 cells**

**A.** Pie chart displaying the number of up- and down-regulated genes in JQ1-treated HepG2 cells compared to DMSO-treated HepG2 cells. Yellow indicates up-regulation, green indicates down-regulation. **B.** Biological processes and GO analysis of down-and up-regulated genes in JQ1-treated HepG2 cells.

**
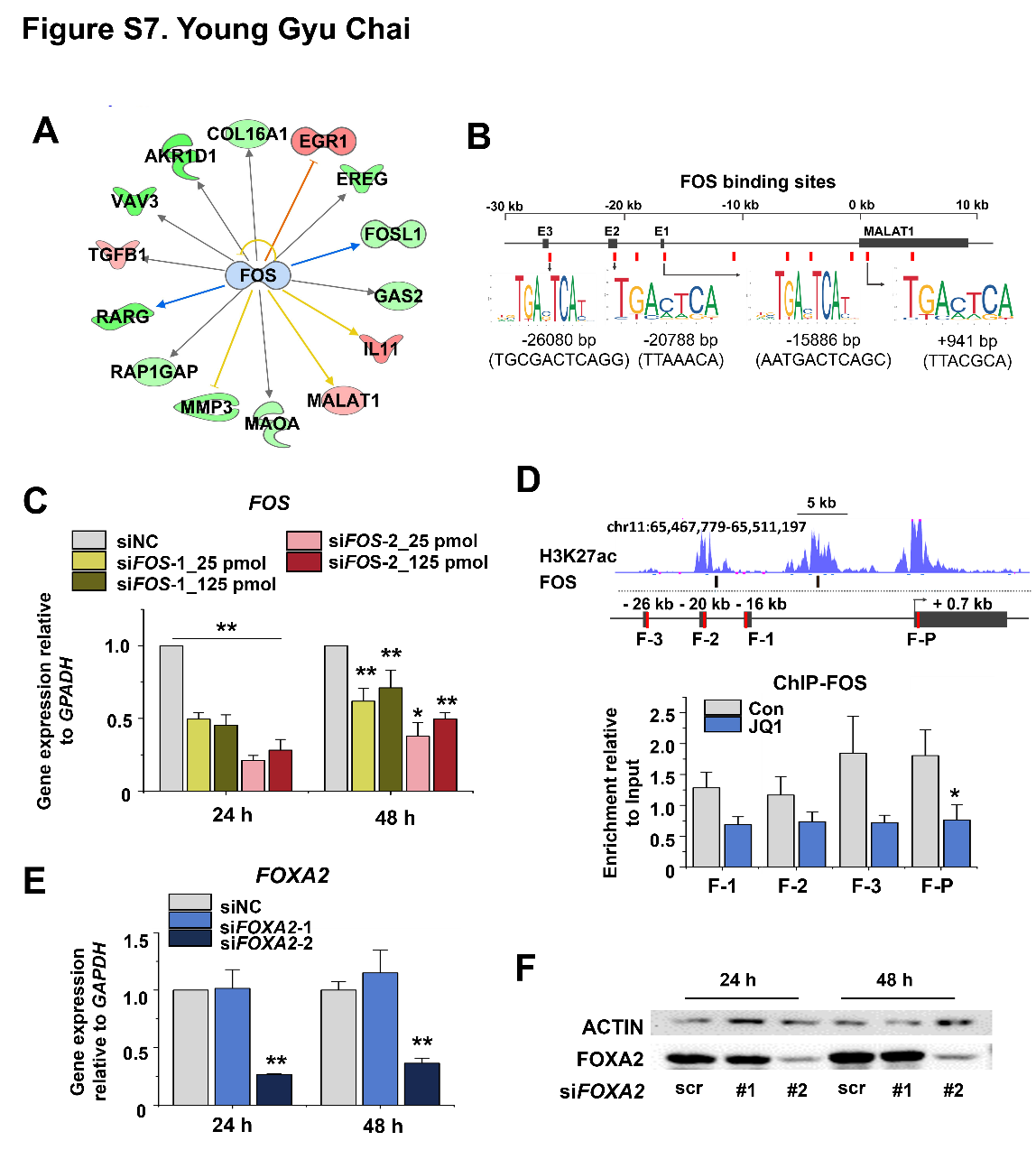
**

**Figure S7. Inhibition of FOS and FOXA2 by RNA interference**

**A.** FOS-centered IPA network analysis of JQ1-treated HepG2 cells. **B.** FOS binding sites (red boxes) in the promoter and putative enhancer regions of the *MALAT1* locus of MCF-7 cells (GSE105734), as identified with JASPAR (<http://jaspar.genereg.net>). **C.** qRT-PCR analysis of *FOS* expression levels in *FOS* siRNA- and scrambled siRNA-treated HepG2 cells. The data represent three independent experiments. The values are the mean ± SD of triplicate wells. *. *P* < 0.05 and **, *p* < 0.01. **D.** Analysis of FOS binding to the *MALAT1* locus. Top panel, H3K27ac enrichment, FOS binding, in HepG2 cells; from published ChIP-seq. The red lines indicate amplicons F-1, F-2, F-3, and F-P, used in our ChIP-qPCR analysis of FOS binding shown in the lower panel. Enrichment was calculated relative to input DNA from three independent experiments. The values are the mean ± SD of triplicate experiments. *, *p* < 0.05 and **, *p* < 0.01. **E.** qRT-PCR analysis of *FOXA2* levels in HepG2 cells treated with *FOXA2* siRNA for 24 or 48 hours. The values are the mean ± SD of triplicate wells. **, *p* < 0.01. **F.** Western blot analysis of FOXA2 protein levels in siRNA-treated HepG2 cells. Scr, scrambled control; #1, si*FOXA2*-1; #2, si*FOXA2*-2.


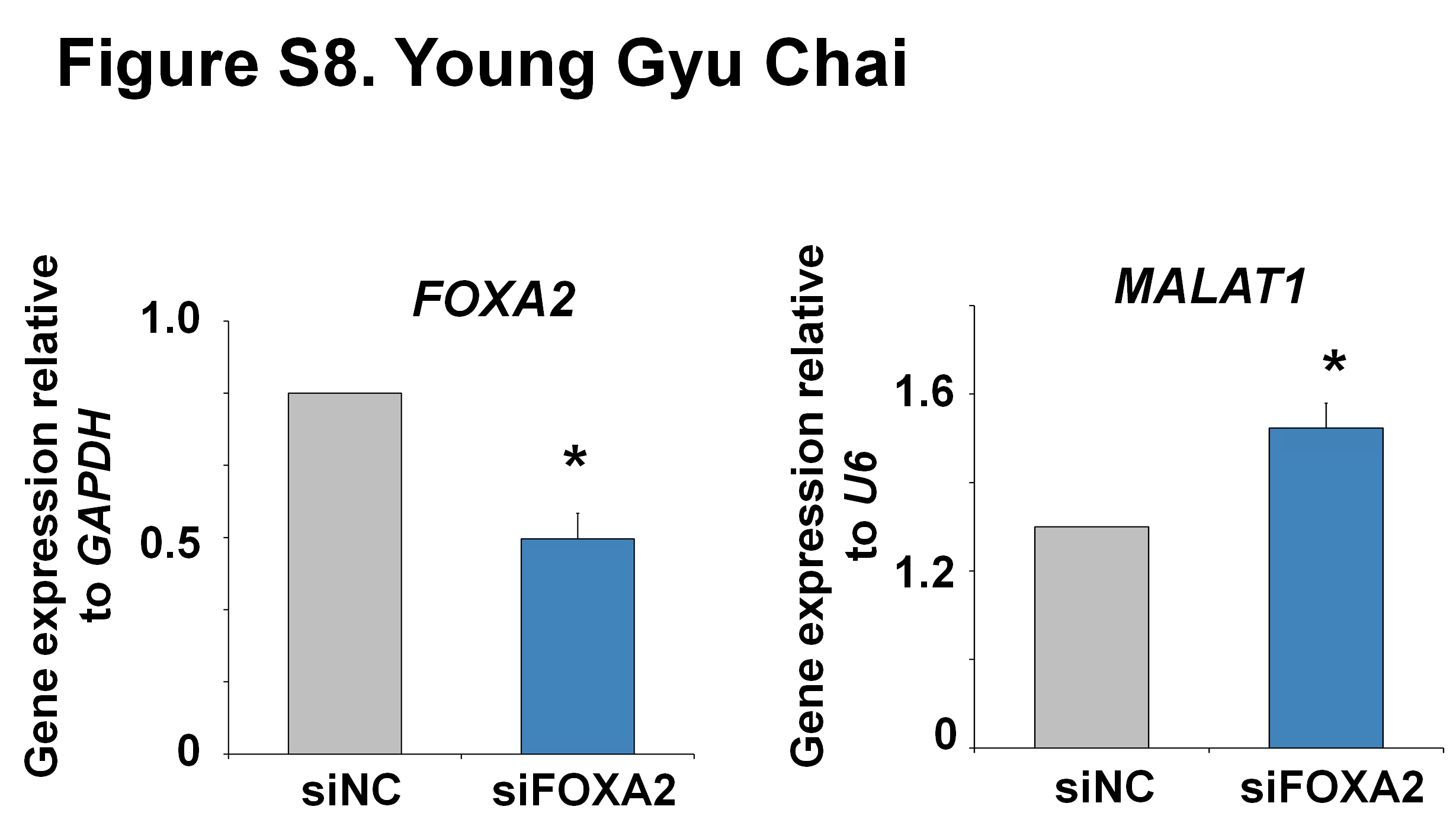


**Figure S8. The effects of FOXA2 inhibition in Huh7 cells**

qRT-PCR analysis of *FOXA2* mRNA and *MALAT1* levels in Huh7 cells treated with *FOXA2* siRNA for 48 hours. The values are the mean ± SD of triplicate wells. *, *p* < 0.05.

**
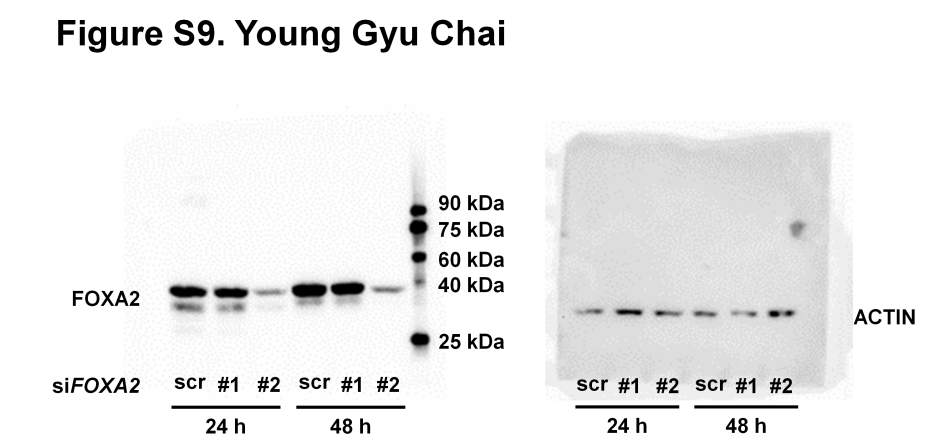
**

**Figure S9. Full-length blots image of FOXA2**

Figure S9 is a full-length blots in Figure S7F. Scr, scrambled control; #1, si*FOXA2*-1; #2, si*FOXA2*-2.


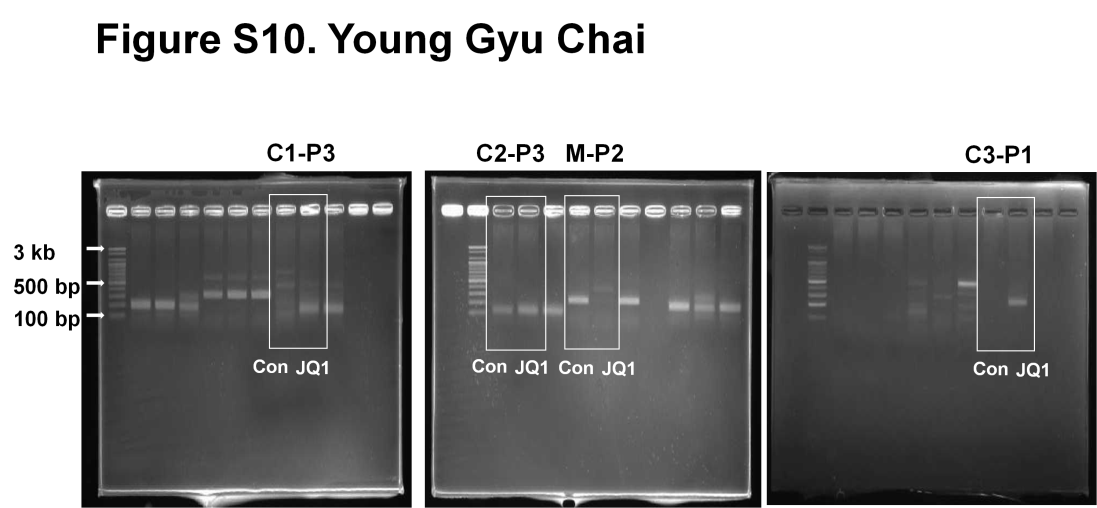


**Figure S10. Full-length gel images of 3C experiment**

Full-length gel image of PCR results in Figure 5A. M, P1, P2, P3, C1, C2, C3, primers.

**Supplementary Tables**

**Table S1. Top 40 significantly up- and down-regulated lncRNAs in JQ1-treated HepG2 cells**

| **Gene Symbol** | **Log_2_ Fold Change** | ***P*-Value** | |
| --- | --- | --- | --- |
| lnc-CHRNB2-3 | 6.519254 | 2.76E-14 | |
| LINC00910 | 3.661033 | 2.79E-175 | |
| lnc-HFE2-2 | 3.427586 | 1.45E-63 | |
| NEAT1 | 3.370876 | 0 | |
| lnc-ING1-1 | 3.191628 | 4.02E-30 | |
| lnc-MRFAP1-2 | 3.161421 | 1.85E-34 | |
| lnc-PARP16-2 | 3.115678 | 3.78E-13 | |
| lnc-RIMKLB-6 | 3.020391 | 2.82E-20 | |
| lnc-SLC5A10-3 | 2.552556 | 2.96E-39 | |
| lnc-SRPRB-3 | 2.426946 | 2.15E-59 | |
| lnc-SERPINB9-3 | 2.406857 | 1.52E-14 | |
| lnc-FAM72D-61 | 2.405085 | 1.61E-08 | |
| lnc-UGDH-3 | 2.398143 | 9.52E-12 | |
| lnc-SERPINB9-5 | 2.372298 | 1.10E-13 | |
| lnc-ACTR2-3 | 2.308755 | 1.25E-09 | |
| lnc-FAM3A-2 | 2.283497 | 1.28E-10 | |
| lnc-PCM1-4 | 2.175978 | 5.08E-65 | |
| lnc-B9D2-2 | 2.138139 | 5.97E-10 | |
| lnc-ERV3-1-2 | 2.119738 | 4.33E-27 | |
| lnc-NES-2 | 2.101143 | 5.48E-12 | |
| lnc-VIP-4 | 2.060864 | 2.30E-09 | |
| lnc-P2RX5-2 | 2.014562 | 6.62E-80 | |
| MALAT1 | 1.981146 | 3.88E-89 | |
| LINC00893 | 1.962256 | 1.28E-07 | |
| lnc-UGDH-4 | 1.939627 | 7.89E-11 | |
| lnc-NUDT3-4 | 1.933234 | 5.09E-10 | |
| lnc-ZNF335-1 | 1.910054 | 7.85E-10 | |
| lnc-MACROD1-1 | 1.864739 | 1.25E-09 | |
| lnc-LYSMD3-1 | 1.838681 | 2.45E-07 | |
| lnc-FBXL2-1 | 1.806862 | 2.74E-19 | |
| lnc-COX19-4 | 1.798748 | 1.11E-09 | |
| LINC01376 | 1.758399 | 5.86E-07 | |
| lnc-LTBP3-2 | 1.73677 | 5.92E-11 | |
| LINC01715 | 1.698092 | 4.17E-08 | |
| lnc-CWC15-1 | 1.692971 | 1.55E-08 | |
| lnc-MOB3C-3 | 1.677353 | 1.17E-10 | |
| lnc-PDLIM7-3 | 1.657919 | 7.60E-07 | |
| lnc-STX5-3 | 1.633245 | 3.02E-06 | |
| lnc-SERPINB1-5 | 1.609233 | 9.86E-23 | |
| RAD51-AS1 | 1.604125 | 5.49E-11 | |
| lnc-TNIK-3 | -5.47491 | | 8.15E-10 |
| lnc-GPAM-2 | -5.09307 | | 1.80E-15 |
| NPSR1-AS1 | -4.43285 | | 1.66E-19 |
| lnc-TH-1 | -4.36084 | | 1.42E-12 |
| LINC00242 | -4.20555 | | 1.18E-12 |
| lnc-DGKK-1 | -4.13578 | | 7.83E-17 |
| lnc-SSX4B-2 | -4.11264 | | 1.00E-13 |
| HNF1A-AS1 | -4.03584 | | 2.13E-25 |
| lnc-FAM84B-15 | -4.01452 | | 2.58E-60 |
| HS1BP3-IT1 | -3.35717 | | 3.56E-05 |
| lnc-OXTR-1 | -3.26643 | | 1.14E-12 |
| LINC01146 | -3.22832 | | 4.37E-38 |
| lnc-LRRC10-4 | -3.21004 | | 1.94E-29 |
| lnc-BRF2-7 | -3.20169 | | 4.67E-16 |
| HAGLR | -3.1234 | | 1.24E-27 |
| lnc-SNORC-2 | -2.89747 | | 7.48E-21 |
| lnc-MTBP-5 | -2.71666 | | 3.04E-13 |
| LINC00261 | -2.63713 | | 9.88E-17 |
| lnc-PDLIM3-6 | -2.58989 | | 7.47E-11 |
| lnc-ICE2-1 | -2.53804 | | 3.56E-14 |
| lnc-NUAK1-7 | -2.49248 | | 4.08E-05 |
| lnc-ANG-1 | -2.4033 | | 7.32E-17 |
| FOXP4-AS1 | -2.39316 | | 8.12E-15 |
| lnc-TNNT2-3 | -2.39229 | | 5.41E-12 |
| lnc-ICE2-3 | -2.3817 | | 6.14E-11 |
| lnc-FREM1-63 | -2.3449 | | 3.19E-09 |
| lnc-PCBD2-1 | -2.34484 | | 6.72E-09 |
| lnc-SOX5-3 | -2.32773 | | 2.23E-16 |
| lnc-HLTF-6 | -2.30831 | | 2.12E-08 |
| lnc-NUAK1-5 | -2.30114 | | 1.15E-12 |
| lnc-PRDM1-6 | -2.28656 | | 6.35E-24 |
| lnc-SEC16B-2 | -2.26358 | | 1.22E-14 |
| lnc-TRIM3-2 | -2.24992 | | 9.60E-15 |
| lnc-GPAM-1 | -2.22936 | | 3.39E-15 |
| lnc-JAML-1 | -2.21429 | | 7.39E-13 |
| lnc-NPY4R2-569 | -2.18637 | | 8.02E-10 |
| PRR7-AS1 | -2.15411 | | 4.57E-10 |
| lnc-CA5A-6 | -2.15049 | | 2.60E-10 |
| lnc-KRCC1-2 | -2.1451 | | 1.80E-55 |
| HNF4A-AS1 | -2.12862 | | 3.34E-23 |

**Table S2. Top 40 significantly up- and down-regulated genes in JQ1-treated HepG2 cells**

| **Gene Symbol** | **Log_2_ Fold Change** | ***P*-Value** |  |
| --- | --- | --- | --- |
| EFR3B | 7.0 | 5.28E-16 |  |
| FOS | 3.9 | 2.31E-10 |  |
| ARRDC4 | 3.7 | 1.53E-21 |  |
| NEAT1 | 3.5 | 0.00E+00 |  |
| TXNIP | 3.2 | 6.29E-98 |  |
| LOC93622 | 3.2 | 3.34E-33 |  |
| EGR1 | 3.1 | 1.26E-53 |  |
| ZNF117 | 3.1 | 3.51E-47 |  |
| MT2A | 2.6 | 4.31E-93 |  |
| ABCA5 | 2.5 | 1.23E-27 |  |
| SERPINB9 | 2.5 | 8.88E-45 |  |
| ZC3H10 | 2.5 | 1.61E-10 |  |
| SRXN1 | 2.4 | 3.23E-215 |  |
| ZSWIM6 | 2.4 | 1.16E-17 |  |
| ERV3-1 | 2.4 | 7.03E-36 |  |
| DHRS2 | 2.4 | 0.00E+00 |  |
| G6PD | 2.3 | 2.02E-46 |  |
| CTGF | 2.3 | 2.44E-67 |  |
| PDE6D | 2.3 | 2.36E-17 |  |
| OSGIN1 | 2.3 | 1.64E-44 |  |
| ZFYVE1 | 2.3 | 6.74E-07 |  |
| MYLIP | 2.3 | 8.63E-11 |  |
| TST | 2.2 | 7.03E-52 |  |
| MTHFR | 2.2 | 2.50E-17 |  |
| SERTAD1 | 2.2 | 1.28E-08 |  |
| MALAT1 | 2.0 | 4.67E-76 |  |
| HEXIM1 | 2.0 | 6.37E-51 |  |
| TMEM169 | 2.0 | 1.66E-07 |  |
| TGFB1 | 2.0 | 2.68E-17 |  |
| CNNM4 | 2.0 | 1.18E-22 |  |
| SERPINB1 | 2.0 | 1.75E-58 |  |
| LGMN | 2.0 | 4.27E-07 |  |
| ORMDL2 | 1.9 | 2.43E-15 |  |
| PTGR1 | 1.9 | 6.52E-06 |  |
| SMIM14 | 1.9 | 2.67E-12 |  |
| SESN3 | 1.9 | 8.80E-13 |  |
| CYR61 | 1.9 | 2.95E-10 |  |
| SFR1 | 1.9 | 1.72E-06 |  |
| NANOS1 | 1.9 | 5.18E-06 |  |
| DOPEY2 | 1.9 | 1.35E-09 |  |
| NR1H4 | -6.0 | 2.62E-34 | |
| CCAT1 | -5.8 | 1.23E-10 | |
| PCYT1B | -5.8 | 7.37E-17 | |
| ODAM | -4.9 | 1.19E-34 | |
| UGT2B4 | -4.8 | 8.29E-15 | |
| MBL2 | -4.8 | 6.22E-20 | |
| INHBE | -4.6 | 3.17E-13 | |
| NPSR1-AS1 | -4.4 | 1.13E-16 | |
| FCGBP | -4.4 | 3.91E-15 | |
| SULT2A1 | -4.2 | 8.50E-64 | |
| PTCHD4 | -4.1 | 2.32E-41 | |
| VAV3 | -4.0 | 1.84E-28 | |
| DGKK | -4.0 | 6.01E-42 | |
| C4BPB | -3.9 | 3.72E-26 | |
| HEPACAM | -3.9 | 5.62E-159 | |
| CPB2 | -3.8 | 9.40E-110 | |
| CPLX2 | -3.7 | 6.76E-102 | |
| SLC2A2 | -3.7 | 1.56E-33 | |
| GPHA2 | -3.6 | 7.60E-15 | |
| UGT2B10 | -3.5 | 2.18E-71 | |
| LINC00176 | -3.5 | 6.07E-66 | |
| GPD1 | -3.4 | 7.68E-34 | |
| NAV2 | -3.3 | 3.82E-33 | |
| SLC38A5 | -3.3 | 1.79E-56 | |
| CACNB4 | -3.3 | 8.81E-30 | |
| KRT23 | -3.3 | 5.04E-16 | |
| ERAP2 | -3.2 | 1.02E-30 | |
| ENPP3 | -3.2 | 2.14E-48 | |
| SSUH2 | -3.2 | 2.32E-19 | |
| SLC7A11 | -3.2 | 6.35E-87 | |
| UBD | -3.2 | 4.20E-20 | |
| ASAH2 | -3.2 | 1.54E-12 | |
| HSD3B7 | -3.2 | 1.24E-36 | |
| PALMD | -3.1 | 8.05E-37 | |
| C19orf80 | -3.1 | 2.89E-11 | |
| KYNU | -3.1 | 6.95E-49 | |
| SGK2 | -3.1 | 1.28E-26 | |
| SERPINA7 | -3.1 | 5.04E-122 | |
| SLC5A9 | -3.1 | 4.90E-22 | |
| SLC30A10 | -3.0 | 4.45E-11 | |

**Table S3. Cloning primer for reporter assay**

| **Enhancer** | **Sequence (5’-3’)** |
| --- | --- |
| MALAT1-E1-F_NheⅠ | ATAGCTAGCGATAAGACCAAGGAAAAAGGCCAGA |
| MALAT1-E1-F_XhoⅠ | ATACTCGAGCAGGTTTTCTCCATGTTCGTCAG |
| MALAT1-E2-F_NheⅠ | ATAGCTAGCCCTGGGCTCTTGTCTTGAGG |
| MALAT1-E2-F_XhoⅠ | ATACTCGAGGGAGTCGGTGACAGAAAGGG |
| MALAT1-E3-F_NheⅠ | ATAGCTAGCCACCTTTGAGTTAGGGCCGA |
| MALAT1-E3-F_XhoⅠ | ATACTCGAGGAAGGCCAGACTAGAAGCCC |

**Table S4. Primer sequences used for qRT-PCR**

| **Gene** | **Forward (5’-3’)** | **Reverse (5’-3’)** |
| --- | --- | --- |
| GAPDH | AAGGTCGGAGTCAACGGATT | CTCCTGGAAGATGGTGATGG |
| U6 | GCTTCGGCAGCACATATACTAAAAT | CGCTTCACGAATTTGCGTGTCAT |
| AREG | GTGGTGCTGTCGCTCTTGATA | CCCCAGAAAATGGTTCACGCT |
| EREG | GTGATTCCATCATGTATCCCAGG | GCCATTCATGTCAGAGCTACACT |
| MAPK3 | CTACACGCAGTTGCAGTACAT | CAGCAGGATCTGGATCTCCC |
| VCAN | GTAACCCATGCGCTACATAAAGT | GGCAAAGTAGGCATCGTTGAAA |
| MALAT1 | CTTTTCTAGGGGATTTCAGG | GCCCACAGGAACAAGTCCTA |
| PVT1 | GCCCCTTCTATGGGAATCACTA | GGGGCAGAGATGAAATCGTAAT |
| DANCR | GCGCCACTATGTAGCGGGTT | TCAATGGCTTGTGCCTGTAGTT |
| HOXD-AS | ATTCGTCTGACTTGGCTCTT | CCTGTTTTGACCTTTTCCTG |
| HNF1A-AS | TCAAGAAATGGTGGCTAT | GCTCTGAGACTGGCTGAA |
| FOS | CGGGCTTCAACGCAGACTA | GGTCCGTGCAGAAGTCCTG |
| EGR1 | GGTCAGTGGCCTAGTGAGC | GTGCCGCTGAGTAAATGGGA |
| ID2 | GCTATACAACATGAACGACTGCT | AATAGTGGGATGCGAGTCCAG |
| JUND | CCTTTCCTCGATCTCGCTCC | AAACAGAAAACCGGGCGAAC |
| FOSL1 | CAGGCGGAGACTGACAAACTG | TCCTTCCGGGATTTTGCAGAT |
| FOXA2 | GGAGCAGCTACTATGCAGAGC | CGTGTTCATGCCGTTCATCC |
| TRIM22 | CTGTCCTGTGTGTCAGACCAG | TGTGGGCTCATCTTGACCTCT |
| HOXD1 | CGGGTCTCACGTCCACTAC | GATGCGGTCTGGAAAGCAC |
| LINC02736 | CCTTGGAAGTGAACTCCCTCA | GGTGGTGAGTGCTGGAATGA |

**Table S5. Primer sequences used for ChIP-PCR**

| **ChIP-PCR** | **Forward (5’-3’)** | **Reverse (5’-3’)** |
| --- | --- | --- |
| B-P | GCTCTGTGGTGTGGGATTGA | TTCGAGAAATCGGAGCAGCA |
| B-1 | GTGGTGGCTGATGAGAACAGA | ACTCTCTGCTGATGCTGCTG |
| B-2 | CCCAGATACCGTTTTCCCGA | GGGTTGACCTAACTTGAGCCT |
| B-3 | AGAAAAAGCCCCATGGCAGA | GCAGTGAGGAGGTGAAAGCA |
| F-1 | GTGGTGGCTGATGAGAACAGA | ACTCTCTGCTGATGCTGCTG |
| F-2 | GAGCTAGGGAGTACAGCGGA | TTTCCCTTCGGGCATCCAAG |
| F-3 | AGAAAAAGCCCCATGGCAGA | GCAGTGAGGAGGTGAAAGCA |
| F-P (F-P2) | GCTCTGTGGTGTGGGATTGA | TTCGAGAAATCGGAGCAGCA |
| X-1 | CCCAGGGACTGTTTCTGGTT | GTGAACAAGCACAGCCTCAC |
| X-2 | ACACAGACCTGCCTCTCTCT | TCCAGAGTTTCCAGTCCGGT |
| X-3 | TAACACCCCCTTATGCCTGG | TAAACCAGTGGCCAGTCTCC |
| X-P | CTGCTCCGGTTCAGAAGGTC | GGGACACACCCAGAAGTGTT |
| F-P1 | CCCAGGTTTCCCAGAGTCCT | GGCCAGCCTATAAGGACAGC |
| F-P3 | TTTTGACAACGCAGCAGAGC | CCGGGCCATTGCTTCATCTA |

**Table S6. Primer sequences used for 3C-PCR**

| **3C-PCR** | **Sequence (5’-3’)** |
| --- | --- |
| C1 | AGGAATGACTCAGCAAGGGC |
| C2 | ACTTGGAAATTTTGTTGGACC |
| C3 | TCTGTCTCCTGTTGCGTGTG |
| P1 | CCACCTGCCCACATAAAGCA |
| P2 | CGTGTAGCTATCAAGGGCCA |
| P3 | AACTACTTTTTGCCTCCCTCACA |
| M | GCCAACACAGTTTGCTCACA |
